# Necrotizing Fasciitis of Scalp and Neck in Neonates

**DOI:** 10.21699/ajcr.v8i3.554

**Published:** 2017-05-01

**Authors:** Sumeet R Dhawan, Pankaj C Vaidya, Jerry R John, Biman Saikia, Ram Samujh, Akshay Saxena, Pratibha D Singhi

**Affiliations:** 1 Department of Paediatrics, Postgraduate Institute of Medical Education and Research, Chandigarh; 2 Department of Plastic Surgery, Postgraduate Institute of Medical Education and Research, Chandigarh; 3 Department of Immunopathology, Postgraduate Institute of Medical Education and Research, Chandigarh; 4 Department of Pediatric Surgery, Postgraduate Institute of Medical Education and Research, Chandigarh; 5 Department of Radiology, Postgraduate Institute of Medical Education and Research, Chandigarh

**Dear Sir,**

Necrotizing fasciitis (NF) has been described with multiple conditions including omphalitis, balanitis, immunodeficiency etc. Back and abdomen are the most commonly affected areas. NF of scalp is rare. We report two neonates with cellulitis of scalp and neck which progressed to necrotizing fasciitis and myositis of neck muscles.

A term 3 kg hospital born neonate presented on day 9 of life with continuous high grade fever since day 2 of life. Baby developed progressive erythema and swelling of scalp, face and periorbital area with small scattered black patches for 5 days (Fig.1A). Complete blood count showed haemoglobin of 13.5 g/dL, total leukocyte count of 26,200/mm3 and platelet count of 71,000/mm3. Coagulogram, cerebrospinal fluid (CSF) analysis, HIV ELISA, blood culture, immunoglobulin profile, T and B-lymphocyte subsets and nitro-blue tetrazolium tests were in normal range. Contrast CT scan showed diffuse skin and subcutaneous thickening of face and neck. Baby was managed with vancomycin, meropenem and clindamycin. He developed clinical deterioration and multiple areas of gangrenous spots surrounded by inflamed edematous skin over right half of face and scalp. Ultrasonography of neck was suggestive of pyomyositis and NF. Extensive debridement of scalp and neck was done. Necrotic material grew Pseudomonas species sensitive only to colistin. Baby was put on intravenous colistin for 6 weeks. Wound improved with fresh granulation tissue on daily dressings with hydrogen peroxide and betadine (Fig.1B). Skin grafting was done subsequently at 2 months of age, but the baby developed secondary infection and septicaemia. Wound culture grew Acinetobactor baumani sensitive to imipenem and meropenem. He succumbed to septic shock and multi organ failure six weeks after skin grafting.

**Figure:**
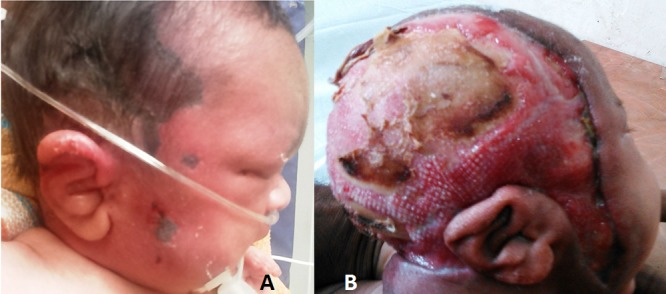
Figure 1: A) Showing cellulitis of face, scalp and neck with areas of cutaneous skin necrosis of case 1. B) showed areas of granulation tissue over the scalp and neck after antibiotic therapy.

A 3-week old full term 3 kg neonate presented with fever, irritability, pus discharge and blackish discoloration of occipital region for 7 days. Illness started with head shaving by the parents. He underwent wide debridement on the day of admission. Pus grew Enterobacter cloacae and CSF revealed Acinetobacter baumannii (sensitive to Meropenem). Baby was treated with meropenem for 6 weeks (titrated according to CSF reports). Complete blood count showed haemoglobin of 9.8 g/dL, total leukocyte count of 23,000/mm3 and platelet count of 3,00,000/mm3. C-reactive protein was elevated. Immune workup including nitro-blue tetrazolium test and CD3/CD19 (CD- Cluster of Differentiation 19) was normal. Child also had eosinophilia (absolute eosinophil count 1170/mm3), IgE levels were 43 IU/ml (range 0-6.6IU/ml). Th17 (helper T cells that produce interleukin-17) and STAT 3 (signal transducer and activator of transcription 3) mutations were normal. The scalp skin grafting was deferred initially because of abnormal wound cultures. Child developed pre-auricular abscess (culture positive for methicillin resistant staphylococcus aureus) at 3 month of age for which she was treated with intravenous vancomycin. The scalp wound improved with time. The child is currently two years of age and healthy and developmentally normal.

NF is a life threatening infection of superficial and deep fascia and subcutaneous tissues and commonly involves abdomen and back.[1,2] Differentiating cellulitis and NF can be difficult. Failure to respond to antibiotics, woody hard feeling, systemic toxicity, bullous lesions and ecchymosis and gangrenous patches suggest NF.[1] In NF, the tissues planes are extremely friable and can be separated even by gloved finger intraoperatively. Involvement of neck and scalp is uncommon.[3] Scalp monitoring with fetal electrodes and intravenous cannulation are associated with increased risk of scalp involvement.[4,5] More than one microbe may be present and during treatment secondary hospital acquired infections can develop. This can prove fatal as occurred in case 1. Mortality varies from 20-60%.[2,6] Prompt initiation of antibiotics and timely debridement are crucial for patient’s survival. Surgical procedures like skin grafting in a neonate is a challenge. Large wound area increases the chance of secondary infection and fluid loss.

## Footnotes

**Source of Support:** Nil

**Conflict of Interest:** None declared

